# Age-dependent microstructural changes of the intervertebral disc: a validation of proteoglycan-sensitive spectral CT

**DOI:** 10.1007/s00330-021-08028-z

**Published:** 2021-05-15

**Authors:** Julian Pohlan, Carsten Stelbrink, Matthias Pumberger, Dominik Deppe, Friederike Schömig, Nils Hecht, Friedemann Göhler, Bernd Hamm, Torsten Diekhoff

**Affiliations:** 1 Department of Radiology, Campus Mitte, Charité - Universitätsmedizin Berlin, Humboldt-Universität and Freie Universität Berlin, Charitéplatz 1, 10117 Berlin, Germany; 2 Spine Surgery, Center for Musculoskeletal Surgery, Radiology, Charité - Universitätsmedizin Berlin, Campus Mitte, Humboldt-Universität zu Berlin, Freie Universität Berlin, Berlin, Germany; 3 Department of Neurosurgery and Center for Stroke Research Berlin (CSB), Radiology, Charité - Universitätsmedizin Berlin, Campus Mitte, Humboldt-Universität zu Berlin, Freie Universität Berlin, Berlin, Germany

**Keywords:** Tomography, X-ray Computed, Spine, Collagen, Chondroitin sulfates, Intervertebral disc

## Abstract

**Objective:**

To analyze the two major components of the intervertebral disc (IVD) in an ex vivo phantom, as well as age-related changes in patients.

**Methods:**

Collagen and chondroitin sulfate were imaged at different concentrations in agar solution. Age-related changes in disc density were retrospectively analyzed in normal-appearing discs in dual-energy computed tomography (DECT) images from a patient cohort with various spinal pathologies (*n* = 136). All computed tomography (CT) scans were acquired using single-source DECT at 80 and 135 kVp with automatic exposure calculation. In 136 patients, the attenuation of normal-appearing discs on collagen/chondroitin maps (cMaps) correlated with the patients’ age with Pearson’s *r* using standardized regions of interest in the anterior anulus fibrosus (AAF) and nucleus pulposus (NP).

**Results:**

DECT collagen mapping revealed concentration-dependent Hounsfield units (HU) of IVD components. For collagen, we found Pearson’s *r* = 0.9610 (95% CI 0.6789–0.9959), *p* = 0.0023 at 120 kVe, and *r* = 0.8824 (95% CI 0.2495–0.9871), *p* = 0.0199 in cMap. For chondroitin sulfate, Pearson’s *r* was 0.9583 (95% CI 0.6603–0.9956), *p* = 0.0026 at 120 kVp, and *r* = 0.9646 (95% CI 0.7044–0.9963), *p* = 0.0019 in cMap. Analysis of normal-appearing IVDs revealed an inverse correlation of density with age in the AAF: Pearson’s *r* = − 0.2294 at 135 kVp (95% CI − 0.4012 to − 0.04203; *p*=0.0141) and *r* = − 0.09341 in cMap (95% CI − 0.2777 to 0.09754; *p* = 0.0003). In the NP, age and density did not correlate significantly at 135 kVp (*p* = 0.9228) and in cMap (*p* = 0.3229).

**Conclusions:**

DECT-based collagen mapping allows microstructural analysis of the two main intervertebral disc components—collagen and chondroitin sulfate. IVD density declines with age, presumably due to a reduction in collagen and chondroitin sulfate content. Age-related alterations of disc microstructure appear most pronounced in the AAF.

**Key Points:**

*• DECT-based collagen mapping allows precise analysis of the two main intervertebral disc components—collagen and chondroitin sulfate.*

*• Intervertebral disc (IVD) density declines with age, presumably due to a reduction in collagen and chondroitin sulfate content.*

*• Age-related alterations of disc microstructure are most pronounced in the anterior anulus fibrosus (AAF).*

**Supplementary Information:**

The online version contains supplementary material available at 10.1007/s00330-021-08028-z.

## Introduction

Intervertebral disc (IVD) degeneration commonly occurs in the aging spine, often leading to degenerative disc disease (DDD) with low back pain [1, 2]. The intervertebral disc connects two vertebral bodies and consists of cartilaginous and fibrous elements—the central nucleus pulposus (NP) and the surrounding anulus fibrosus [3]. Collagen and proteoglycans such as chondroitin are the main biochemical components of the disc and are thought to play a significant role in the development of DDD [4, 5].

Microstructural alteration of IVDs may occur before obvious morphological change becomes apparent and is attributed to reorganization of the extracellular matrix [6]. Importantly, imaging features of degeneration such as disc bulging, disc protrusion, and annular fissure also occur in asymptomatic individuals and increasingly with advanced age [7]. Magnetic resonance imaging (MRI) is the current reference standard for assessment of spinal conditions involving soft tissue with excellent morphological depiction of the IVD and early degenerative changes [8]. MRI provides additional information when sequences such as glycosaminoglycan chemical exchange saturation transfer (gagCEST) are used [9]. However, several contraindications limit the use of MRI in patients, with an ongoing debate about cardiac implants [10, 11].

Another diagnostic modality to assess for discogenic pain would be provocative discography, with contrast application after placing a needle in the IVD [12, 13]. This procedure has been proven to be a safe and accurate diagnostic procedure [14]. Imaging findings of DDD are not only prevalent in symptomatic individuals but also highly prevalent among asymptomatic individuals [7]. Therefore, provocative discography, performed according to the International Association for the Study of Pain (IASP) criteria, remains the most specific procedure to diagnose discogenic low back pain [12, 15].

Dual-energy computed tomography (DECT) may facilitate depiction of the characteristic properties of collagen with relatively high attenuation of the densely packed fibers and a relatively low effective atomic number, thus improving visualization of tendons, ligaments, and other collagen-rich structures [16]. Recent studies suggest that DECT is also useful in depicting the morphology and microstructural changes of IVDs [17, 18]. Additionally, virtual non-calcium (VnCa) maps have been shown to increase diagnostic performance and confidence for depicting lumbar disc herniation as compared with standard CT [19]. However, a systematic analysis of quantitative and qualitative aspects has yet to be conducted.

Therefore, the aim of this study was to analyze major components of the IVD in a phantom setup using DECT and to determine whether the results may be transferred to age-related changes of IVD microstructure in patients.

## Material and methods

### Ethics approval and informed consent

This study was approved by the local ethics board under EA1/230/19. Patient consent was waived given the retrospective nature of the analysis.

### Phantom setup

Collagen (Merck) and chondroitin sulfate (Merck) were separately added at five different concentrations to small vials, each containing 2 ml of heated 2% agar solution, i.e., control agar (0)–67.8 mg collagen (1)–125.7 mg collagen (2)–252.0 mg collagen (3)–495.0 mg collagen (4)–1005.0 mg collagen (5) and control agar (0)–67.4 mg chondroitin sulfate (1)–125.3 mg chondroitin sulfate (2)–251.0 mg chondroitin sulfate (3)–502.0 mg chondroitin sulfate (4)–1011.0 mg (5). All vials were thoroughly mixed. The phantom was surrounded by water during imaging.

### Patient recruitment

**Fig. 1:**
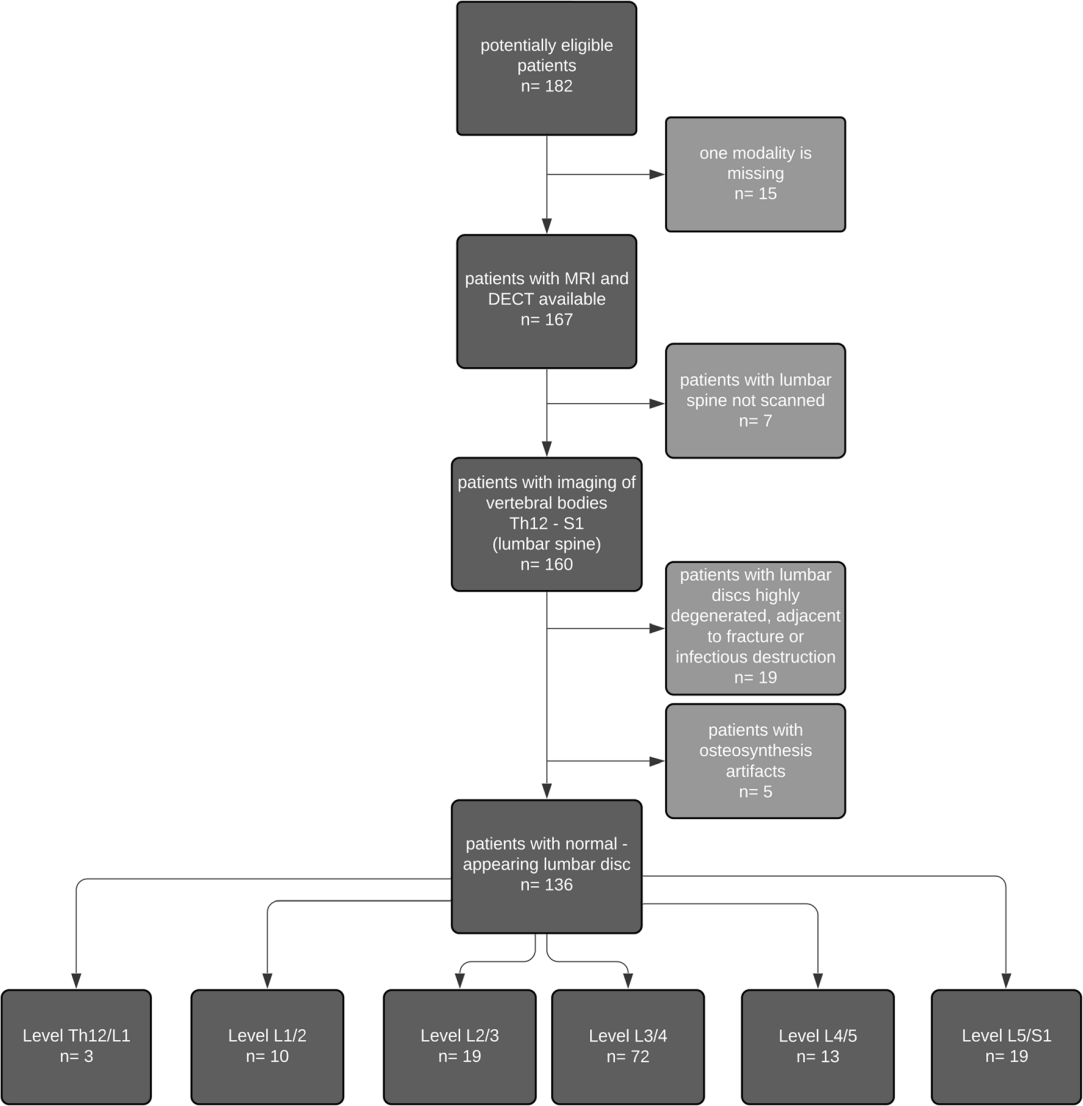
Flow chart of patient selection. We retrospectively included patients from four separate studies who underwent CT and MRI for different indications. Specifically, we included patients who underwent CT-guided infiltration (*n* = 14), patients with axial spondyloarthritis (*n* = 75), patients with osteoporotic vertebral fractures (*n* = 68), and patients with suspected septic spondylodiscitis (*n* = 25), totaling *n* = 182. All reasons for exclusion are provided

We retrospectively included patients who underwent a DECT scan of the lumbar spine from Nov. 2014 to Feb. 2020. Patients without a recent MRI scan of the lumbar spine at least two months prior to or after the DECT scan were excluded. For analysis, one normal-appearing IVD per patient was identified based on morphological appearance in both MRI and DECT datasets. Disc abnormalities such as bulging, disc segment adjacent to vertebral fracture, discitis, spondylitis, or severe artifacts (e.g., due to metal implants) resulted in the exclusion of the corresponding intervertebral disc. If eligible, L3/4 was chosen for further measurement. Otherwise, we selected L2/3 or L4/5 and so on for analysis, prioritizing a level close to L3/4 (Fig. 1). Only one disc was included per patient. If no normal lumbar disc was present, the scan was excluded from analysis.

### DECT scanning

CT images were acquired in a conventional single-source CT scanner (Aquilion ONE Vision, Canon Medical Systems) equipped with a 320-row detector to allow repeated scanning without table movement. Dual-energy datasets were obtained using the technique of sequential acquisition of two volumes (135 kVp and 80 kVp). The phantom experiment was performed with ascending tube current (10, 20, 30, 40, 50, 70, 90, 110, 130, 150 mA). Gantry rotation time was 0.275 s with a changeover time of 0.5 s, resulting in an overall scanning time of 1.05 s per scan. Patients were imaged in a prone or supine feet-first position. Images were acquired using automatic exposure calculation (AEC) with a standard deviation of 12 Hounsfield units (HU), and the wide-volume technique was applied if the scan range exceeded 16 cm.

### Postprocessing

Dual-energy datasets were reconstructed with 0.5 mm slice thickness using a medium soft tissue kernel without beam hardening compensation and image-based iterative reconstruction (AIDR-3D, standard level). CT datasets were then further postprocessed at the CT console to generate collagen/chondroitin maps (cMaps) using a collagen-specific gradient of 1.1 and applying a three-material-decomposition algorithm.

### Ex vivo phantom measurement

Images were transferred to a workstation with a high-resolution monitor. Using Horos (Version 4.0, Horos project, based on OsiriXTM open source), we defined circular regions of interest (ROIs); all analyses were performed with 3 mm averaged slice thickness in axial reformation.

For the phantom setup, 371 mm^2^ standardized ROIs with a diameter of 21.7 mm were placed in representative areas of the vial containing collagen (*n* = 196) or chondroitin sulfate (*n* = 175) at different concentrations. Three hundred eighteen ROIs were analyzed for the phantom experiment (excluding 53 ROIs for internal control, e.g., H_2_O).

### Patient measurement

A total of 136 IVDs from 136 patients were included in the analysis. One 30-mm^2^ standardized ROI with a diameter of 6.2 mm each was placed in the NP and in the AAF, in oblique axial reformations of 3 mm slice thickness both in 135-kVp images and in cMaps. A total of 544 ROIs, four in each patient, were analyzed for age correlation. Measured HU were exported using the “Export ROI” plugin and converted to an Excel file (Microsoft, Office 365 MSO).

Similarly, MRI standardized ROIs (10 mm^2^ for ROIs in the AAF and 20 mm^2^ for ROIs in the NP) were placed at sagittal short-tau intensity recovery (STIR) or turbo-inversion recovery-magnitude (TIRM) sequences at 4 mm, and exported for further analysis. Intensity values were normalized by subtraction of intensities from 20 mm^2^ ROIs placed in the paravertebral musculature.

### Statistics

Descriptive statistics were computed for the phantom and patient measurements. We performed Pearson’s correlation analysis of the disc density data, as criteria for linearity were fulfilled. The paired *t* test was used to compare densities measured in male and female patients. Age groups for density measurements were set to < 29, 30–39, 40–49, 50–59, 60–69, 70–79, > 80. Analyses were performed using ANOVA. All statistics were conducted with IBM SPSS (Version 25.0) and GraphPad Prism (Version 8).

## Results

### Phantom measurement

**Fig. 2:**
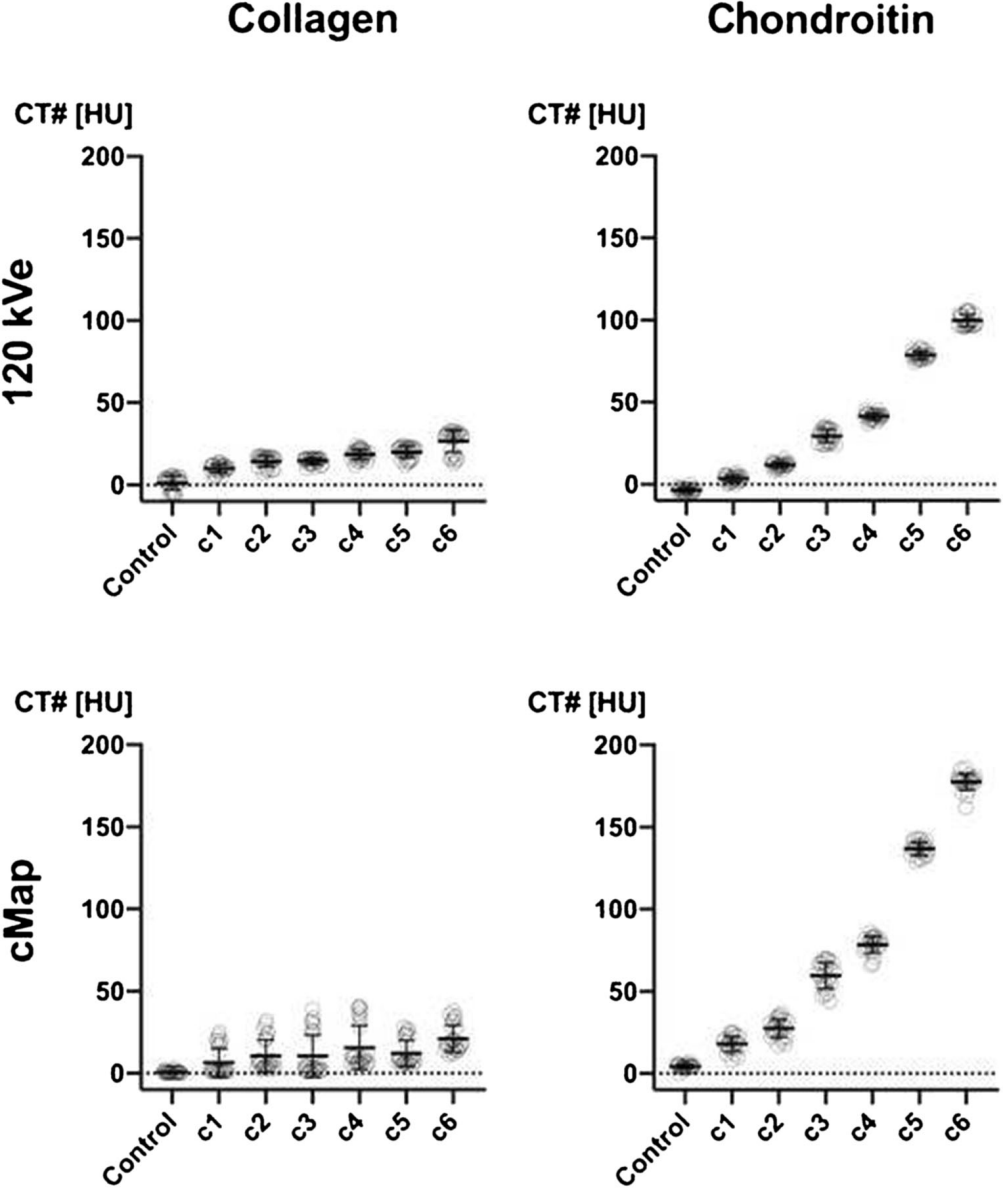
Results of density measurements in the phantom. Attenuation in HU for collagen and chondroitin sulfate at different concentrations measured in 120 kVe-equivalent and cMap reconstructions. Attenuation shows a steady increase with the concentration, which is more pronounced for chondroitin sulfate compared with collagen. *cMap = collagen/chondroitin map; HU = Hounsfield unit*

Analysis of different concentrations of collagen solution yielded a high correlation of density and mass at 120 kVe with *r* = short-tau intensity recovery 0.9610 (95% CI 0.6789 to 0.9959), *p* = 0.0023 (Fig. 2). For cMaps, we found a correlation with *r* = 0.8824 (95% CI 0.2495 to 0.9871), *p* = 0.0199. The density gradient (135/80 kV) was 1.01 for collagen and 0.8 for chondroitin sulfate.

Chondroitin sulfate displayed a significant concentration-dependent density in conventional CT scans with a positive linear correlation at 120 kVe with Pearson’s *r* = 0.9583 (95% CI 0.6603 to 0.9956), *p* = 0.0026, and *r* = 0.9646 (95% CI 0.7044 to 0.9963), *p* = 0.0019, in cMaps, respectively.

### Patient measurement

Among the 182 patients who underwent DECT, MRI identified 136 patients with morphologically normal lumbar discs (see Fig. 1). The mean age of the patients included was 54.91 years (SD 18.90). The proportion of female patients was at 54.4%. CT scans were performed with a mean dose-length product (DLP) of 476.83 ± 366.17 mGy*cm and with a computed tomography dose index volume (CTDI_vol_) of 13.95 ± 8.85 mGy.

The NP showed a density of 84.6 HU ± 11.8 at 135 kVp and 98.0 HU ± 34.1 in cMaps, the AAF 90.3 HU ± 11.0 at 135 kVp and 107.1 HU ± 34.1 in cMaps. Differences in density of the AAF compared to the NP were significant both at 135 kVp (paired *t* test; *p* = < 0.0001) and in cMaps (*p* = 0.0004).

One-way ANOVA identified significantly different disc density of the levels Th12/L1 and L5/S1 for both AAF and NP compared with the levels in between. Upon exclusion of Th12/L1 and L5/S1, there was no significant difference between IVD levels L1-L5 with regard to density at 135 kVp and in cMaps (Table 1).

**Table 1:** Level-dependent density in human intervertebral discs

Levels included	Th12–S1		L1–L5	
Number of patients	*n* = 136		*n* = 114	
Localization	NP	AAF	NP	AAF
135 kVp	*p* = 0.0494	*p* = 0.2355	*p* = 0.2724	*p* = 0.5937
cMap	*p* < 0.0001	*p* = 0.0046	*p* = 0.9937	*p* = 0.4805

As shown in the left side of the table, statistical analysis with one-way ANOVA identified significantly different disc density of the Th12/L1 and L5/S1 levels for both nucleus pulposus and anterior anulus fibrosus compared with the levels in between. Upon exclusion of Th12/L1 and L5/S1, shown in the right side of the table, there was no significant difference between intervertebral disc levels L1–L5 with regard to density at 135 kVp and in cMaps

*NP* nucleus pulposus, *AAF* anterior anulus fibrosus

### Correlation with age

**Fig. 3:**
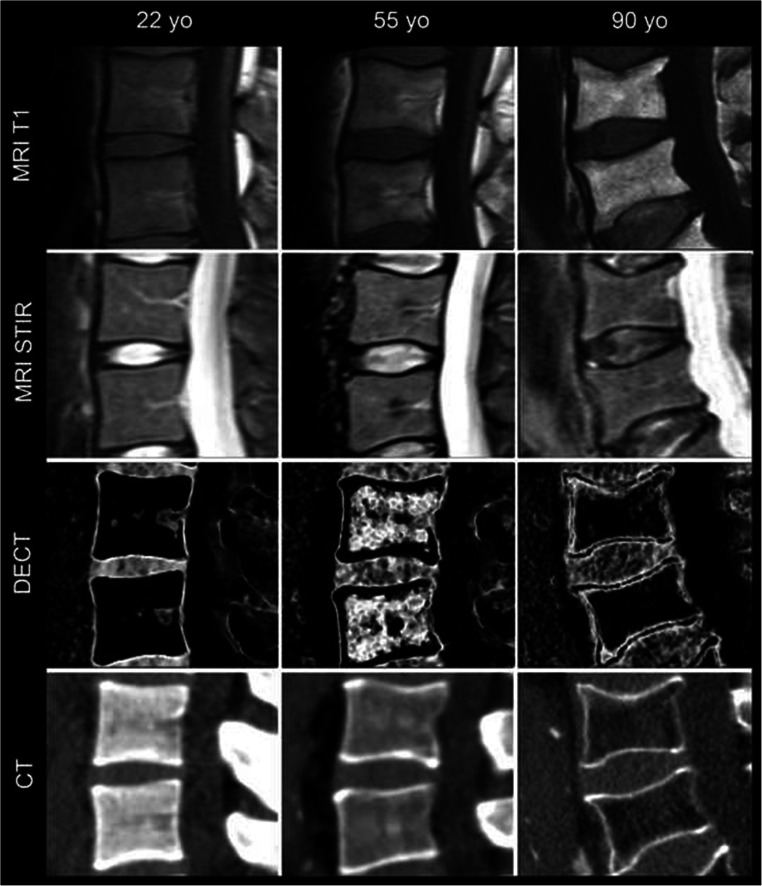
Morphology of intervertebral discs in different age groups. Age-dependent morphology of intervertebral discs in three patients of different ages: first column, aged 22: density of NP, 89.5 HU, and AAF, 100.5 HU at 135 kVp; NP, 113.8 HU, and AAF, 132.1 HU in cMap; second column, aged 55: density of NP, 77.1 HU, and AAF, 101.4 HU at 135 kVp; NP, 99.6 HU, and AAF, 107.0 HU in cMap; and third column, aged 90 years: density of NP, 66.9 HU, and AAF, 76.6 HU at 135 kVp; NP, 56.7 HU, and AAF, 85.0 HU in cMap. Images show sagittal DECT cMaps with corresponding sagittal CT scans; below, T1-weighted MR images and T2-weighted STIR images are shown. *DECT = dual-energy computed tomography; MRI = magnetic resonance imaging*

**Fig. 4:**
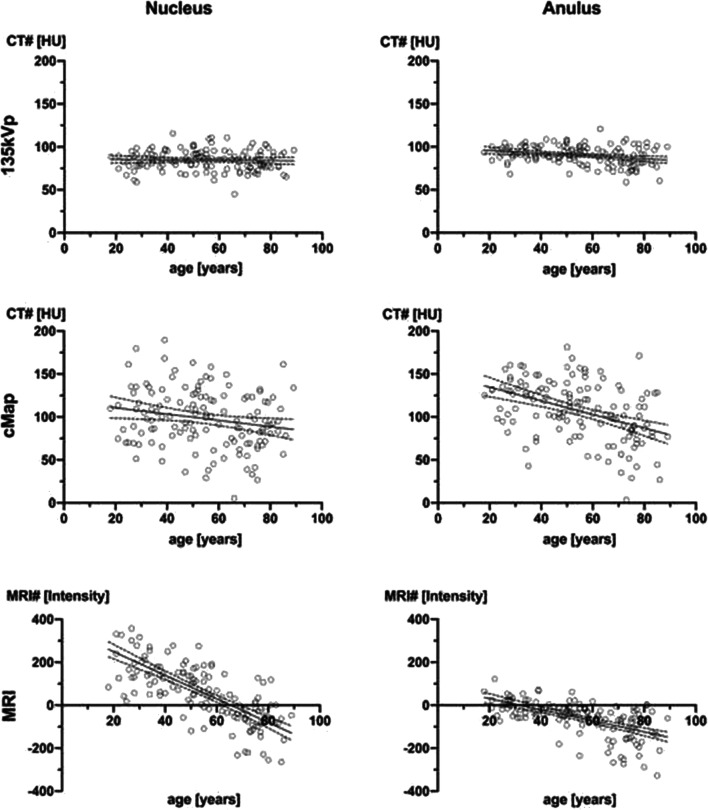
Age-dependent cMap density and MR intensity of human intervertebral discs. Density of normal-appearing intervertebral disc (IVD) compartments measured in conventional 135-kVp images and cMaps in correlation with patient age. There is no significant correlation in 135-kVp images for the NP (*p* = 0.65), whereas cMaps show significant correlation (*p* = 0.02; Pearson’s *r* = − 0.2). Conversely, 135-kVp images show a significant correlation of HU with age in the AAF (*p* = 0.002; *r* = − 0.27) and an even stronger correlation in the cMaps (*p* < 0.001; *r* = − 0.43), suggesting an added value of these reconstructions. The MRI intensities of IVD compartments are inversely correlated with age. Relative intensities were calculated as IVD ROIs in the sagittal STIR sequence normalized by subtraction of ROIs in the paravertebral musculature. STIR intensity values significantly correlated with age at the AAF with *p* = < 0.0001; *r* = − 0.60 (95% CI − 0.70 to − 0.48), and at the NP with *p* = < 0.0001; *r* = − 0.74 (95% CI − 0.81 to − 0.66). *STIR = short-tau inversion recovery*

There were morphological changes in aging subjects (Fig. 3). Measurement at 135 kVp revealed a nonsignificant negative linear correlation of HU and age with Pearson’s *r* = − 0.044 (CI − 0.211 to 0.125) and *p* = 0.609 for the NP. For the AAF, there was a weak negative linear correlation with *r* = − 0.217 (CI − 0.371 to − 0.050) and *p* = 0.011. Analysis of cMap densities of the NP yielded a significant negative linear correlation of HU and age with Person’s *r* = − 0.201 (CI − 0.357 to − 0.034) with *p* = 0.019. In cMaps, correlation for the AAF was *r* = − 0.418 (CI − 0.548 to − 0.269) with *p* = < 0.0001 (Fig. 4).

Linear logistic regression of the ROI measurements in the AAF in correlation with age yielded a slope of − 0.14 (95% CI − 0.25 to − 0.032; *p* = 0.011) at 135 kVp and − 0.77 (95% CI − 1.06 to − 0.488; *p* = < 0.0001) in cMaps. The NP had a nonsignificant slope of − 0.027 (95% CI − 0.13 to 0.077; *p* = 0.61) at 135 kVp and − 0.37 (95% CI − 0.68 to − 0.063; *p* = 0.019) in cMaps. cMap density in the AAF was analyzed according to the respective age group, with significant differences in the ANOVA with *p* < 0.0001. Density of the NP in different age groups yielded no significant differences (*p* value = 0.30).

Gender differences in density analyzed between male and female discs were nonsignificant. At 135 kVp, the male AAF displayed a mean density of 91.1 HU ± 13.5 HU compared with the female AAF density of 90.2 HU ± 11.1 with *p* = 0.85, whereas the male NP had 84.4 HU ± 12.9 and the female NP 85.4 HU ± 10.2 with *p* = 0.56. In the cMaps, the male AAF density was 112.7 HU ± 33.9 and female AAF 102.6 HU ± 35.4 with *p* = 0.099, while the male NP had a mean density of 102.0 HU ± 35.2 and the female NP a mean of 96.4 HU ± 34.9 with *p* = 0.379.

STIR intensity values significantly correlated with age at the AAF with *p* = < 0.0001, *r* = − 0.60 (95% CI − 0.70 to − 0.48), and at the NP with *p* = < 0.0001, *r* = − 0.74 (95% CI − 0.81 to − 0.66) (see Fig. 4).

Correlation of MRI STIR intensity values with cMap densities yielded a significant correlation for the AAF with *p* = 0.0002, *r* = 0.32 (95% CI 0.16 to 0.46), and was nonsignificant for the NP with *p* = 0.41, *r* = 0.07 (95% CI − 0.098 to 0.24) (see supplementary Figure S1).

## Discussion

This is the first study aimed at analyzing intervertebral disc components in a phantom setup using DECT and transferring the experimental data to age-related changes in IVD microstructure in patients.

Our phantom experiments show a concentration-dependent density of the discal components collagen and chondroitin sulfate on DECT imaging. However, chondroitin sulfate contributes more strongly to the densities measured in both conventional CT images and cMaps. Patient-based analysis indicates a steady age-dependent decline of density in the AAF and NP, which is better depicted by cMaps compared to conventional CT images. Age-dependent density loss appears to be more pronounced in the AAF than in the NP.

Our results suggest that DECT is capable of assessing the proteoglycan content of the IVD and might therefore be sensitive to disc degeneration and other conditions that affect microstructure and proteoglycan content. However, aging is a significant factor that requires consideration in assessing disc pathologies on DECT images. These results reflect the physiological loss of major disc components in normal-appearing IVDs, which needs to be separated from pathological degeneration and disc destruction.

The age-dependent loss of both collagen and chondroitin sulfate we observed in the AAF and NP is strongly supported by a recent biochemical analysis of human IVDs [20]. Experimental data in goat discs suggest that proteoglycan loss may contribute significantly more markedly to extracellular matrix reduction in disc degeneration than collagen [6]. This supports our notion that the observed age-related changes in our study population are mostly driven by proteoglycan content and less so by changes in collagen concentration.

When analyzing the age-related change in disc density, we controlled for the impact of different IVD levels on density. Both level Th12/L1 and L5/S1 were found to have significantly different densities compared with levels L1–5. We attribute this in part to the fact that only three IVDs of level Th12/L1 were analyzed. The higher density of L5/S1 may be related to the fact that this disc carries more weight than other levels. Moreover, in our patient population, most discs at this level were examined in very young patients, resulting in bias due to young age. Repeat correlation analysis of age-related density excluding levels Th12/L1 and L5/S1 proved a significant correlation between age and disc density in the AAF only.

Our data clearly show an age-dependent loss of IVD fluidity, as seen in the inverse correlation of STIR intensity of both AAF and NP in discs of different age. This is a known result of a concurrent loss of collagen/proteoglycans and water content predominantly in the NP [21, 22]. Additional linear regression analysis revealed a significant reduction of density in the AAF per year of age, which was close to 1 HU per year in the cMap. Recent data also confirm the role of DECT in the analysis of lumbar disc degeneration; however, the investigators used a different postprocessing technique that is nonspecific to disc material [18].

While others have analyzed IVDs on DECT VNCa maps, our approach is to use cMaps for IVD visualization. VNCa virtually subtracts calcium from the image, providing indirect measurements of the collagen content similar to conventional CT. cMaps, however, measure the target substances directly by their dual-energy absorption. We previously reported on a patient with spondylodiscitis in whom we demonstrated pronounced loss of signal in cMaps reflecting the extent of disc destruction [23]. We were also able to show that DECT can be sensitive to microstructural disc injuries in patients with vertebral compression fractures [17]. More recently, we were able to show the IVD morphology on cMaps with excellent correlation between DECT and MRI in disc herniation [24]. These results support the accuracy of IVD imaging in various spine pathologies. The current study lays the groundwork for future investigation into the potential of disc imaging with collagen- and proteoglycan-sensitive DECT reconstruction. Also, follow-up imaging using DECT may play a role in the evaluation of microstructural IVD regeneration, while assessing new therapeutic targets [25]. In our data, no gender-related differences in density of the IVD were noted.

While we took care in planning and conducting our phantom experiments, some limitations have to be discussed. First, the preparation of collagen for the in vitro phantom setup yields a heterogeneous fluid, resulting in greater variance of the acquired data compared with chondroitin sulfate. Still, the authors consider these results relevant, as an impact of chondroitin sulfate on DECT cMap density was not reported previously. Second, the analysis of age-dependent changes in IVD microstructure stems from a retrospective study design, which limits the conclusions that can be drawn from these data. This retrospective analysis included CT scans acquired for various reasons (e.g., fracture, septic discitis, and axial spondyloarthritis); however, a certain inclusion bias might be present. Also, all intervertebral discs were morphologically assessed using both MRI and DECT images.

### Conclusion

DECT imaging of IVDs is an emerging imaging option for patients with various spine pathologies. Our results reveal proteoglycans as a major contributor to disc density in DECT imaging besides collagen. Age-related changes in IVD microstructure can be depicted by DECT, and dedicated reconstructions (cMaps) may yield supplementary information compared to conventional CT images.

## Supplementary Information

ESM 1 (DOCX 293 kb)

## Abbreviations

AAF: Anterior anulus fibrosus
AEC: Automatic exposure calculation
CI: Confidence interval
cMap: Collagen/chondroitin map
CT: Computed tomography
DDD: Degenerative disc disease
DECT: Dual-energy computed tomography
HU: Hounsfield unit
IVD: Intervertebral disc
MRI: Magnetic resonance imaging
NP: Nucleus pulposus
ROI: Region of interest

## Declarations

### Guarantor

The scientific guarantor of this publication is Julian Pohlan.

### Statistics and biometry

One of the authors (TD) has significant statistical expertise. No complex statistical methods requiring further support by biometry were necessary for this paper.

### Study subjects or cohorts overlap

Some study subjects have been previously reported; i.e., the patients with osteoporotic vertebral fractures were differently analyzed in another study published in this journal under DOI 10.1007/s00330-018-5963-4. Please compare with the following section in the manuscript: “Specifically, we included patients […] with osteoporotic vertebral fractures (n = 68) […].”

### Methodology

• retrospective (with reference to the intervertebral disc analysis in patients); the experimental setup was prospective in nature

• observational and experimental

• performed at one institution
