# Supplementary material for: Age-dependent microstructural changes of the intervertebral disc: a validation of proteoglycan-sensitive spectral CT
Source: Eur Radiol. 2021 May 15;31(12):9390–8. doi: 10.1007/s00330-021-08028-z (PMC8589800; doi:10.1007/s00330-021-08028-z)

**Fig. S1 – Axial DECT cMaps and densities**

DECT cMap in axial reformation showing an age-dependent loss of density in both the AAF and NP density in three patients (similar to the patients presented in fig. 3).


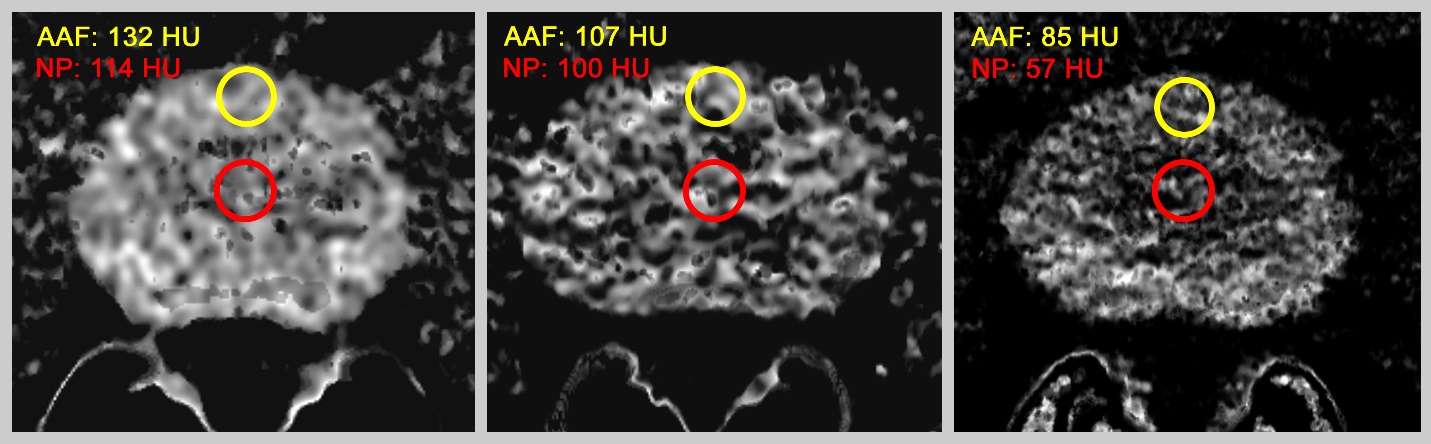


**Fig. S2 – correlation of DECT and MRI**

Correlation analysis of DECT cMap density and MR intensity values of the IVD. Relative MRI STIR intensity values were calculated from ROIs in the AAF and NP of the IVD normalized to the paravertebral musculature. There was a significant correlation between AAF density in DECT cMaps and relative STIR intensity with p = 0.0002; r = 0.32 (95% CI 0.16 to 0.46). A non-significant correlation was noted in the NP with p = 0.41; r = 0.07 (95% CI -0.098 to 0.24). These data reflect the different biochemical composition of different regions of the IVD.


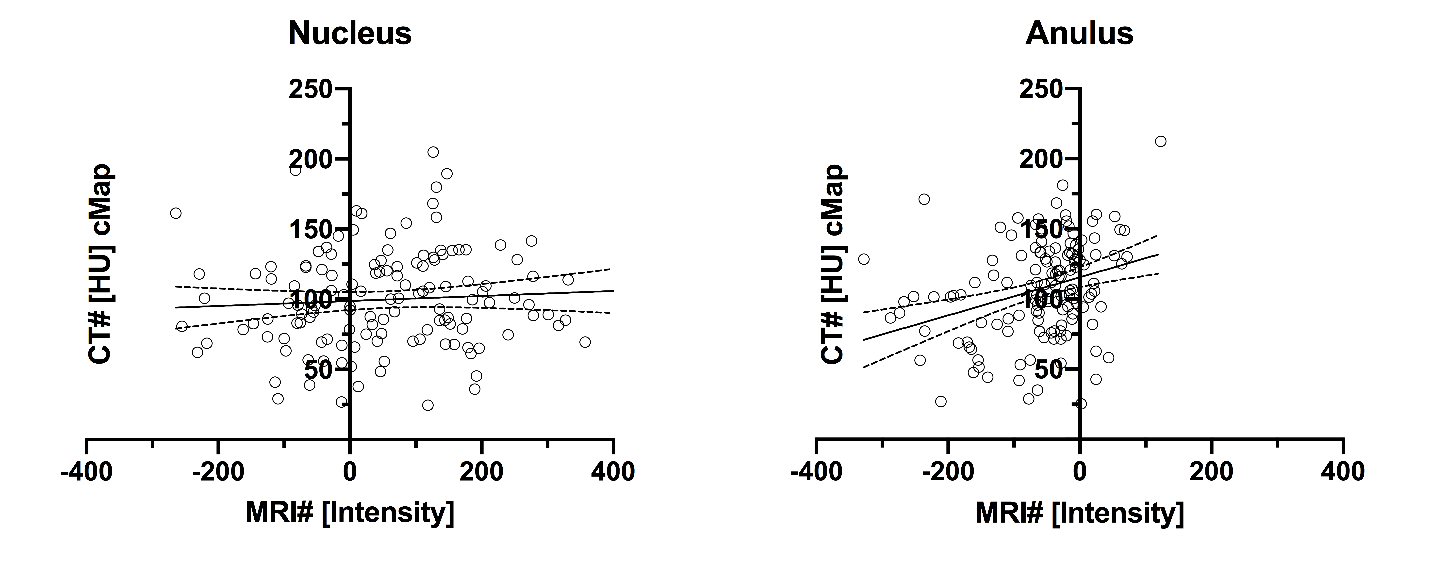

Supplement: Supplementary file 1 — (DOCX 293 kb) [file 330_2021_8028_MOESM1_ESM.docx]
